# Microbial cytosine deaminase is a programmable anticancer prodrug mediating enzyme: antibody, and gene directed enzyme prodrug therapy

**DOI:** 10.1016/j.heliyon.2022.e10660

**Published:** 2022-09-16

**Authors:** Ashraf S.A. El-Sayed, Nabil Z. Mohamed, Marwa A. Yassin, Mahmoud M. Amer, Reyad El-Sharkawy, Nesma El-Sayed, Mostafa G. Ali

**Affiliations:** a Enzymology and Fungal Biotechnology Lab, Botany and Microbiology Department, Faculty of Science, Zagazig University, 44519, Egypt; b Botany and Microbiology Department, Faculty of Science, Benha University, Benha, 13518, Egypt

**Keywords:** Cytosine deaminase, 5-fluorcytosine, 5-fluoruracil, ADEPT, GDEPT

## Abstract

Cytosine deaminase (CDA) is a non-mammalian enzyme with powerful activity in mediating the prodrug 5-fluorcytosine (5-FC) into toxic drug 5-fluorouracil (5-FU), as an alternative directed approach for the traditional chemotherapies and radiotherapies of cancer. This enzyme has been frequently reported and characterized from various microorganisms. The therapeutic strategy of 5-FC-CDA involves the administration of CDA followed by the prodrug 5-FC injection to generate cytotoxic 5-FU. The antiproliferative activity of CDA-5-FC elaborates from the higher activity of uracil pathway in tumor cells than normal ones. The main challenge of the therapeutic drug 5-FU are the short half-life, lack of selectivity and emergence of the drug resistance, consistently to the other chemotherapies. So, mediating the 5-FU to the tumor cells by CDA is one of the most feasible approaches to direct the drug to the tumor cells, reducing its toxic effects and improving their pharmacokinetic properties. Nevertheless, the catalytic efficiency, stability, antigenicity and targetability of CDA-5-FC, are the major challenges that limit the clinical application of this approach. Thus, exploring the biochemical properties of CDA from various microorganisms, as well as the approaches for localizing the system of CDA-5-FC to the tumor cells via the antibody directed enzyme prodrug therapy (ADEPT) and gene directed prodrug therapy (GDEPT) were the objectives of this review. Finally, the perspectives for increasing the therapeutic efficacy, and targetability of the CDA-5-FC system were described.

## 1 Introduction

Cytosine deaminase (CDA) (E.C 3.5.4.1) is an amidohydrolase that catalyzes the deamination of cytosine into uracil and ammonia (Kilstrup et al., 1989; Danielsen et al., 1992; Mullen, 1994; Mullen et al., 1994). CDA has been distributed in microorganisms with potentiality to convert the non-toxic 5-fluorocytosine (5-FC) into toxic 5-fluorouracil (5-FU) (Polak and Scholar, 1975; Charles et al., 1957). The activity of CDA for deaminating the 5-FC into 5-FU was firstly described by Polak and Scholar (1975). 5-Fluorouracil and its oral prodrug capecitabine are the most efficient chemotherapeutic regimens in cancer therapy (Longley et al., 2003; Miura et al., 2010; Vermorken et al., 2007; Argilés et al., 2020), for treatment of several neoplasms such as head and neck squamous cell carcinoma, gastrointestinal, adenocarcinoma and uterine cervix (Chen et al., 2019; Sakai et al., 2019; Argilés et al., 2020). Recently, 5-FU in combination with vascular endothelial growth factor (VEGF) inhibitors (Ghafouri-Fard et al., 2021) has been recognized as efficient approach for cancer therapy. The activity of 5-FU is basically attributed to the affinity to block the activity of cellular thymidylate synthase (TS), thus, preventing the DNA replication (Peters et al., 1994), in addition to the inhibition of RNA synthesis by integration with the RNA (Silverstein et al., 2011). Nevertheless, the short half-life time, quite lack of selectivity and the drug resistance of 5-FU are the major challenges that limits the clinical applications of this enzyme (Takahashi et al., 2014). Thus, manipulating the toxicity and targetability of 5-FU by the prodrug 5-FC-mediated CDA is one of the most sophisticated anticancer approach. The CDA-5-FC has been recognized as a targeted/directed therapy with little side effects, higher and antiproliferative efficiency, than the traditional anticancer therapies such as chemotherapy and radiotherapy (DeVita et al., 1993; Mullen et al., 1994).

The CDA-5-FC prodrugs enzyme mediated therapy has been developed as one of the most successful cutting-edge technologies for cancer therapy (Karjoo et al., 2013). Recently, the strategy of gene-directed enzyme prodrug therapy (GDEPT) “suicide gene therapy” using CDA has been established in order to overcome the chemotherapeutic side effects of the traditional approaches (Karjoo et al., 2013). The antiproliferative activity this prodrug mediated system elaborates from the over activity of uracil pathway in tumor cells compared to normal cells (Heidelberger et al., 1957). Recently, several strategies have been focused on survey of CDA with high turnover and catalytic efficiency, less antigenicity from various microorganisms for activation of the prodrug 5-FC into active 5-FU for selectively targeting the tumor cells with no effect on normal cell (Eastman and Perez, 2006). Bacterial cytosine deaminase (CDA) has received an immense attention over the yeasts enzymes, however, the catalytic efficiency of yeast CDA for deaminating of 5-FC have been noticed to be higher than bacterial CDA (Polak and Scholar, 1975). Nevertheless, the conformational/structural and thermal stabilities of CDA is the major challenge that limits their broad spectrum clinical applications (John and Morris, 2002).

**Figure 1:**
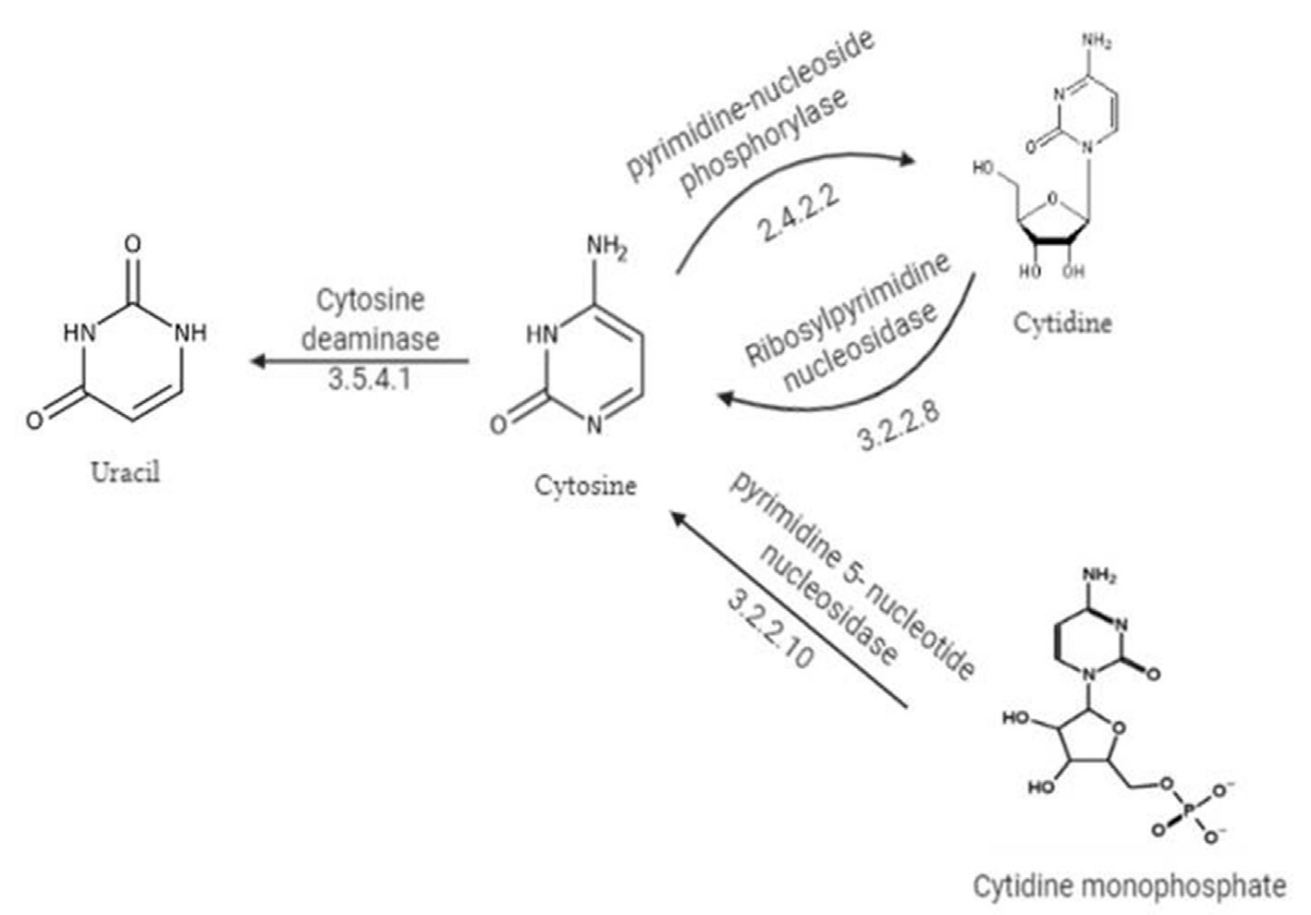
Proposed metabolic pathway of cytosine.

The activity of CDA for conversion of cytosine or its analog 5-fluorocytosine was firstly reported by Polak and Scholar (1975) (Figure 1). Also, the potential antifungal activity of 5-FC was attributed to the activity of intrinsic CDA, deaminating the non-toxic 5-FC into toxic 5-FU (Grunberg and Titsworth, 1963). The superior therapeutic efficiency of 5-FC elaborates from its higher solubility in water and smaller molecular size, facilitating its quickly diffusion in the body (Daneshmend and Warnock, 1983). Practically, the 5-FU has a broad bioactivity range against different pathogenic microorganisms including *Candida*, *Cryptococcus*, *Phialophora*, *Cladosporium* and *Aspergillus* in addition to the antiprotozoal activity; *Leishmania* and *Acanthamoeba* (Polak and Scholar, 1975; Polak et al., 1985; Abdel-Fatah et al., 2021, ​Ali et al., 2015). CDA is a non-mammalian enzyme, with extraordinary clinical affordability for mediating the conversion of 5-FC into 5-FU upon external infusion (Diasio et al., 1978; Williams et al., 1981). Practically, the intestinal microbes could be the main source for the 5-fluorouracil in human cells (Harris et al., 1986).

**Figure 2:**
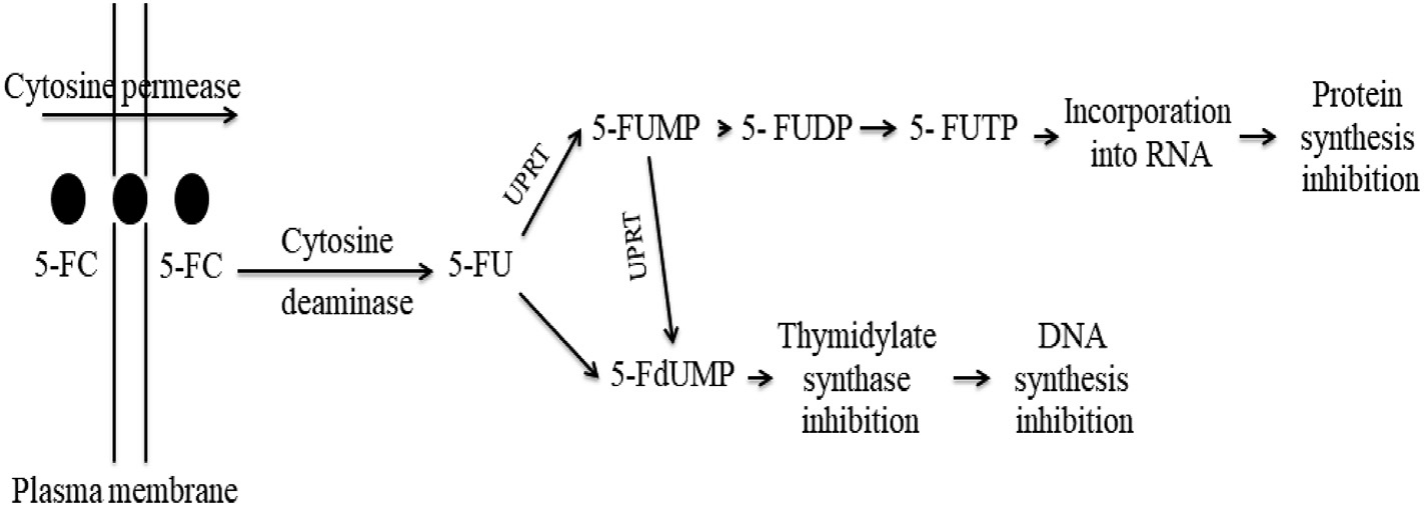
Proposed pathway of 5-FC metabolism and its action mechanism. Uridine phosphoribosyltransferase (UPRT), 5-fluorouridine monophosphate (FUMP), 5- Fluorouridine diphosphate (FUDP), 5-fluorouridine triphosphate (FUTP) and 5-fluorodeoxyuridine monophosphate (FdUMP) as adopted by Vermes et al. (2000).

Cytosine has been metabolized by pyrimidine-nucleoside phosphorylase (E.C 2.4.2.2) and CDA (E.C 3.5.4.1) into cytidine and uracil (Figure 1). Ribosylpyrimidine nucleosidase (E.C 3.2.2.8) and pyrimidine 5-nucleotide nucleosidase (E.C 3.2.2.10) hydrolyze cytidine and cytidine monophosphate to produce cytosine (Kandeel and Al-Taher, 2020). The antiproliferative activity of 5-FC has been reported to be maximized by combining with many antifungal azole agents such as fluconazole and ketoconazole especially against colorectal carcinoma. Practically, 5-FC displayed an efficient antifungal activity that could be due to the presence of CDA, that being a selective strategy for human fungal pathogen treatment (Vermes et al., 2000). Cytosine and 5-FC are competitive nucleotides for transportation across plasma membrane by cytosine permease, that followed by subsequent hydrolysis by CDA into uracil or 5-FU, respectively (Polak and Grenson, 1973). The conversion of 5-FC to 5-FU is only dependent on the presence of active CDA, some fungi do not possess an active CDA that were described as 5-FU resistant fungi (Polak, 1977). In fungi, after 5-FC uptake and conversion to 5-flurouracil, two metabolic mechanisms are implemented to fulfill the antifungal activity of 5-fluorouracil (Figure 1). Firstly, uridine phosphoribosyltransferase (UPRT) converts 5-FU to 5-fluorouridine monophosphate (FUMP), and to 5-fluorouridine diphosphate (FUDP), and finally 5-fluorouridine triphosphate (FUTP) (Waldorf and Polak, 1983; Maamoun et al., 2021). The produced FUTP inhibits the synthesis of cellular proteins by incorporation with fungal RNA replacing the uridylic acid (UTP), hence, altering the acylation of amino acid in tRNA, subsequently disturbing the sequence of amino acid and final block to protein synthesis (Waldorf and Polak, 1983). Secondly, 5-FU is metabolized by uridine 5′-monophosphate pyro-phosphorylase to produce 5-fluorodeoxyuridine monophosphate (5-FdUMP) (Waldorf and Polak, 1983), strongly affects DNA biosynthesis by inhibiting thymidylate synthetase (TS) as the main source of thymidine (Dtaszo et al., 1978). Subsequently, 5-fluoro-deoxyuridine monophosphate inhibits the fungal DNA synthesis and in turn their cellular replication (Figure 2) (Polak and Scholar, 1975; Waldorf and Polak, 1983; Vermes et al., 2000; Vandeputte et al., 2012).

**Figure 3:**
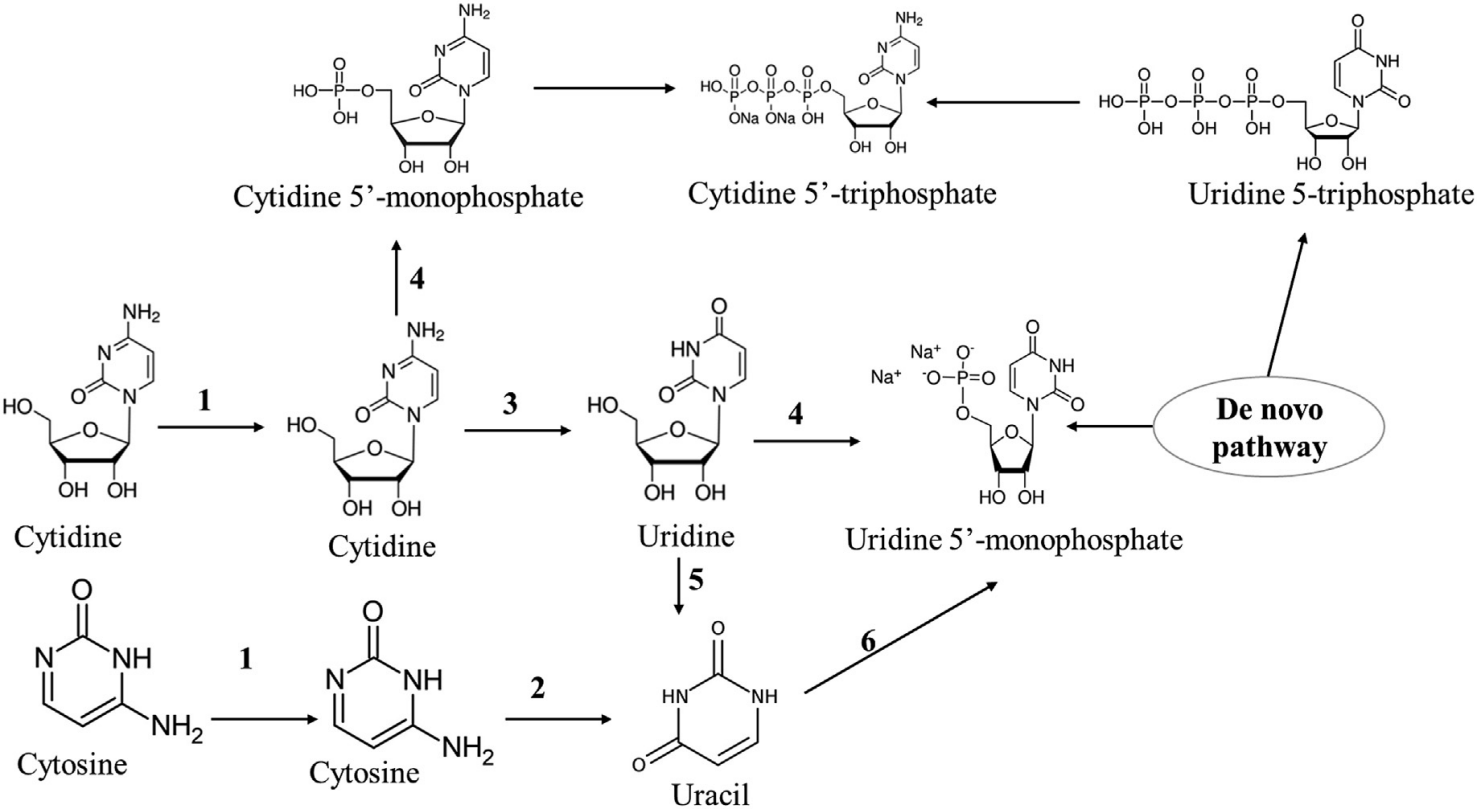
Salvage pathway of cytosine and cytidine in *S. typhimurium* and *S. cerevisiae*. Transporters of the cytosine and cytidine to cell (1), Cytosine deaminase CDA (2), Cytidine deaminase (3), uridine-cytidine kinase (4), uridine phosphorylase (5), uracil phosphoribosyl transferasrase (6).

Salvage pathways are one of the profound cellular assimilatory pathways in most of microbes for synthesis of cytosine and its derivatives from the non-essential nutrients that usually requires less energy than the *de novo* pathways (Yao et al., 2005). The *de novo* pathway for pyrimidine synthesis is an universal pathway for pyrimidine and purine nucleotides synthesis (O'Donovan and Neuhard, 1970). The pyrimidine pathway (Figure 3) was firstly explored in *Salmonella typhimurium* (Beck et al., 1972), different bacterial (Ovrebo and Kleppe, 1973; Rima and Takahashi, 1977; Jyssum and Jyssum, 1979), and fungal species (Chakraborty and Loring, 1960; Polak and Scholar, 1975). The pyrimidine salvage pathway was initiated by importing the nucleotides to the cell, followed by metabolic assimilation of these bases (Kern et al., 1990; Yao et al., 2005). The processes of pyrimidine transportation to the cytosol involves; uracil permease (Chevallier, 1982; Jund et al., 1988), 2- Purine-cytosine transporters that transport cytosine to the cell (Schmidt et al., 1984), and 3- Uridine permease that transports uridine into the cell (Wagner et al., 1998). Unlike the human cells, microorganisms have an active CDA, catalyzing the conversion of 5-FC (non-toxic prodrug) into 5-FU (toxic anticancer drug) (Yao et al., 2005). Thus, expression of CDA in combination with uracil phosphoribosyl-transferase in tumor cells increases the cellular sensitivity to 5-FC and 5-FU (Erbs et al., 2000; El-Kalyoubi et al., 2021, ​El-Mekawy et al., 2010, ​El-Sayed et al., 2010, ​El-Sayed et al., 2011).

### 1.1 Sources and biochemical properties of CDA

Cytosine deaminase has been widely distributed in various bacterial and fungal isolates (Mullen et al., 1992; Hamaji et al., 2007) as listed on Table 1. Bacterial CDA has received a much attention comparing to the fungal one. Although, the higher conformational stability of bacterial CDA, fungal CDA exhibited a higher catalytic efficiency for converting of 5-FC into 5-FU (Polak and Scholar, 1975). Bacterial CDA was mainly intracellular enzyme (Katsuragi et al., 1986), while fungal CDA was reported as intracellular (Yu et al., 1991) and extracellular enzyme (Zanna et al., 2011; Zanna et al., 2012). The biochemical properties of CDA from different microorganisms have been listed in Table 2. Apparently, CDA from the different microbial sources shares the same biochemical properties. For example, the optimum pH was ranged from 6.5 to 9.0, pH stability was ranged from pH 7.0 to pH 10.0, with an optimal temperature at ∼45 °C for the enzyme from bacterial and fungal sources, suggesting the proximity of enzyme conformational structures and catalytic identities. Structurally, the molecular subunit structure was appeared to be ranged from 32.0–48.0 kDa, with two subunits “35 kDa and 46 kDa”, i.e. heterodimeric identity (Katsuragi et al., 1986). Cytosine deaminase from *S. typhimurium* is a homotetrameric enzyme, with molecular mass 54.0 kDa for each subunit (West et al., 1982), and heterodimeric with mass about 78.0 kDa and 149 kDa (Kim and Yu, 1998). However, CDA from *A. fumigatus* was reported as a monomer of 32 kDa (Yu et al., 1991).

**Table 1:** Microbial species producing cytosine deaminase.

Organism	Species	References
Bacteria	*E. coli*	(Katsuragi et al., 1986; Kilstrup et al., 1989; Danielsen et al., 1992; Hussein and Al-Baer, 2018)
	*Serratia marcescens*	(Sakai et al., 1975a; Esders and Lynn, 1985)
	*Pseudomonas aureofaciens*	(Sakai et al., 1975b; Esders and Lynn, 1985)
	*Salmonella typhimurium*	(West et al., 1982)
	*Alcaligenes denitrijicans*	(Kim et al., 1987)
	*Arthrobacter* sp. Jll	
	*Chromobacterium violaceum* YK 391	(Kim and Yu, 1998)
	*Aerobacter aerogenes* IFO 3319	([Bibr bib127][Bibr bib128])
	*Aerobacter cloacae* lAM 1221	
	*Agrobacterium tumefaciens* lAM B-26-1	
	*Arthrobacter simplex* AKU 0620	
	*Arthrobacter pascens* IFO 12139	
	*Bacillus subtilis* IFO 3026	
	*Bacillus sphaericus* IFO 3525	
	*Escherichia coli* K12 AKU 0005	
	*Micrococcus flavus* AKU 0502	
	*Pseudomonas chlororaphis* IFO 3904	
	*Pseudomonas cruciviae* IFO 12047	
	*Pseudomonas ovalis* IFO 3738	
	*Pseudomonas solanacearum* IFO 12510	
	*Bacillus megaterium* AKU 0203	
Yeast	Bakers' yeast	(Ipata et al., 1971; Esders and Lynn, 1985; Katsuragi et al., 1989)
Fungi	*Aspergillus parasiticus*	(Zanna et al., 2012)
	*Aspergillus fumigatus* IFO 5840	(Yu et al., 1991)
	*Aspergillus niger*	(Zanna et al., 2011)
	*Aspergillus flavus*	
	*Aspergillus tamarii*	
	*Aspergillus ustus*	
	*Aspergillus fumigatus*	(El-Sayed et al., 2021)

**Table 2:** Cytosine deaminase properties from different microorganisms.

Microorganism	M. wt. (kDa/subunit)	Optimal pH	pH stability	Optimal temperature (°C)	Specific activity (Unit/mg of protein)	Enzyme substrate	K_m_ (mM)	References
*S. typhimurium*	54	7.30–7.50		45–50		Cytosine	0.74	(West et al., 1982)
*Escherichia coli*	50					Cytosine	0.22	(Porter and Austin, 1993)
*Escherichia coli*						Cytosine	0.2	(Mahan et al., 2004)
						5- flurocytosine	3.3	
*Escherichia coli*	35 & 46	9.0	9.0–10.0	50		Cytosine		(Katsuragi et al., 1986)
*Alcaligenes denitrijicans*	37	8.0–9.0	12.0		4.0	Cytosine	0.1	(Kim et al., 1987)
*Chromobacterium violaceum* YK 391‏	78	7.5		40–45		Cytosine	1.55	(Kim and Yu, 1998)
						5- flurocytosine cytidine	5.52	
						5- methylcytosine	10.4	
							67.2	
*Escherichia coli*	48	8.5	7.5–9.0	45–60	9.0			(Hussein and Al-Baer, 2018)
*Pseudomonas aureofaciens*	45	8.0–9.0				Cytosine		(Sakai et al., 1975b)
						5- methylcytosine		
*S. marcescens*	72	8.0	7.0–9.0			Cytosine		(Sakai et al., 1975a)
*Aspergillus parasiticus*		7.2		40–45		Cytosine	0.19	(Zanna et al., 2012)
						5- flurocytosine	0.30	
*Aspergillus fumigatus* IFO 5840	32	7.0		35		Cytosine	2	(Yu et al., 1991)
						5- flurocytosine	6.5	
						5- methylcytosine	36	
Baker's yeast	41	7.5	7.5	30–40		Cytosine	3.1	(Katsuragi et al., 1989)
						5- flurocytosine	1.2	
Baker's yeast	34	6.5	5.0–9.0			Cytosine	2.5	(Ipata and Cercignani, 1978)
						5- methylcytosine	2.5	
*Aspergillus fumigatus*	48	7.0		37		Cytosine	0.08	(El-Sayed et al., 2021)

From the kinetics studies of CDA from different sources towards different substrates, CDA from *Alcaligenes denitrijicans* (Kim et al., 1987), *A. parasiticus* (Zanna et al., 2012), and *E. coli* (Porter and Austin, 1993), displayed the highest catalytic affinity for cytosine and 5-FC. Interestingly, the affinity of bacterial (Kim et al., 1987; Mahan et al., 2004) and fungal (Zanna et al., 2012) CDA for deaminating cytosine and 5-fluorocytosine being higher than CDA from yeast's, as revealed from the *K*_*m*_ and *K*_*cat*_ values. For example, the value of *K*_*m*_ for *Alcaligenes denitrijicans* CDA was lower than that of baker's yeast by approximately 31 folds, revealing the higher affinity of bacterial enzymes for cytosine (Ipata and Cercignani, 1978; Katsuragi et al., 1989).

The catalytic identity of CDA from different microorganisms has been chemically mapped. The CDA from *E. coli* was strongly inhibited by thiols suicide compounds; *p*-chloromercuribenzoate, *p*-chloromercuriphenylsulfonate and mersaryl acid, approving the cysteine-dependent identity of enzyme (Katsuragi et al., 1986), despite *p*-mercuribenzoate had no inhibitory effect on CDA from baker yeast (Ipata et al., 1971). Yu et al. (1998) reported that amino acids; tryptophan and cysteine residues are located beside the active sites of the *A. fumigatus* CDA. To investigate the identity of the enzyme active sites, the enzyme was incubated with some chemical modifiers, cysteine, histidine, and arginine residues were found to be engaged on the active sites of yeast CDA (Yergatian et al., 1977). The CDA from *Chromobacterium violaceum* was strongly inhibited by mercury compounds such as *p*-chloromercuri-benzoate that being similar to CDA from *A. fumigatus* (Yu et al., 1991), *Pseudomonas aureofaciens* (Sakai et al., 1975a, Sakai et al., 1975b), *E. coli* (Katsuragi et al., 1986), yeast (Yergatian et al., 1977; Katsuragi et al., 1989), and *Arthrobacter* sp (Yeeh et al., 1985). However, CDA from *Serratia marcescens* did not affected by *p*-chloromercuribenzoate suggesting the lack of implementation of thiols on active domains of this enzyme (Nakanishi et al., 1989). CDA from *C. violaceum* (Kim and Yu, 1998) and *A. fumigatus* (Yu et al., 1991) was strongly inhibited by chloroamine T, ensuring the thiols dependence of this enzyme. The activity of CDA was strongly inhibited by Pyridoxal-5-phosphate ensuring the presence of lysine residues in the catalytic domains of the enzyme *C. violaceum* (Kim and Yu, 1998). The suicide inhibitors of serine residue namely phenylmethylsulfonyl fluoride (PMSF) (Kojima et al., 1994; Choi and Kim, 1995), and histidine suicide analogue diethylpyrocarbonate (DEPC) exhibiting a mild effect on *C. violaceum* CDA (Kim and Yu, 1998), unlike to the strong effect on *A. fumigatus* enzyme (Yu et al., 1991). Kim and Yu (1998) reported that the active site domains of CDA from *C. violaceum* are mainly thiols containing amino acids “methionine and cysteine”. The metalloproteinic identity and dependence of CDA on Fe^2+^ has been reported for the purified enzyme from *A. fumigatus* (Yu et al., 1991), *E. coli* (Katsuragi et al., 1986) and yeast (Katsuragi et al., 1989) as revealed from the dialysis against *O*-phenanthroline. Similar results were reported for CDA from *S. marcescens* (Sakai et al., 1975a, Sakai et al., 1975b) and *P. aureofaciens* (Sakai et al., 1975a, Sakai et al., 1975b). The activity of cytosine deaminase from *E. coli* was strongly inhibited by HgCl_2_ and CuSO_4_, suggesting the implementation of SH on the enzyme active sites (Hussein and Al-Baer, 2018) and *A. parasiticus* (Zanna et al., 2012).

### 1.2 Amino acids sequences, active sites domains and crystal structure

**Figure 4:**
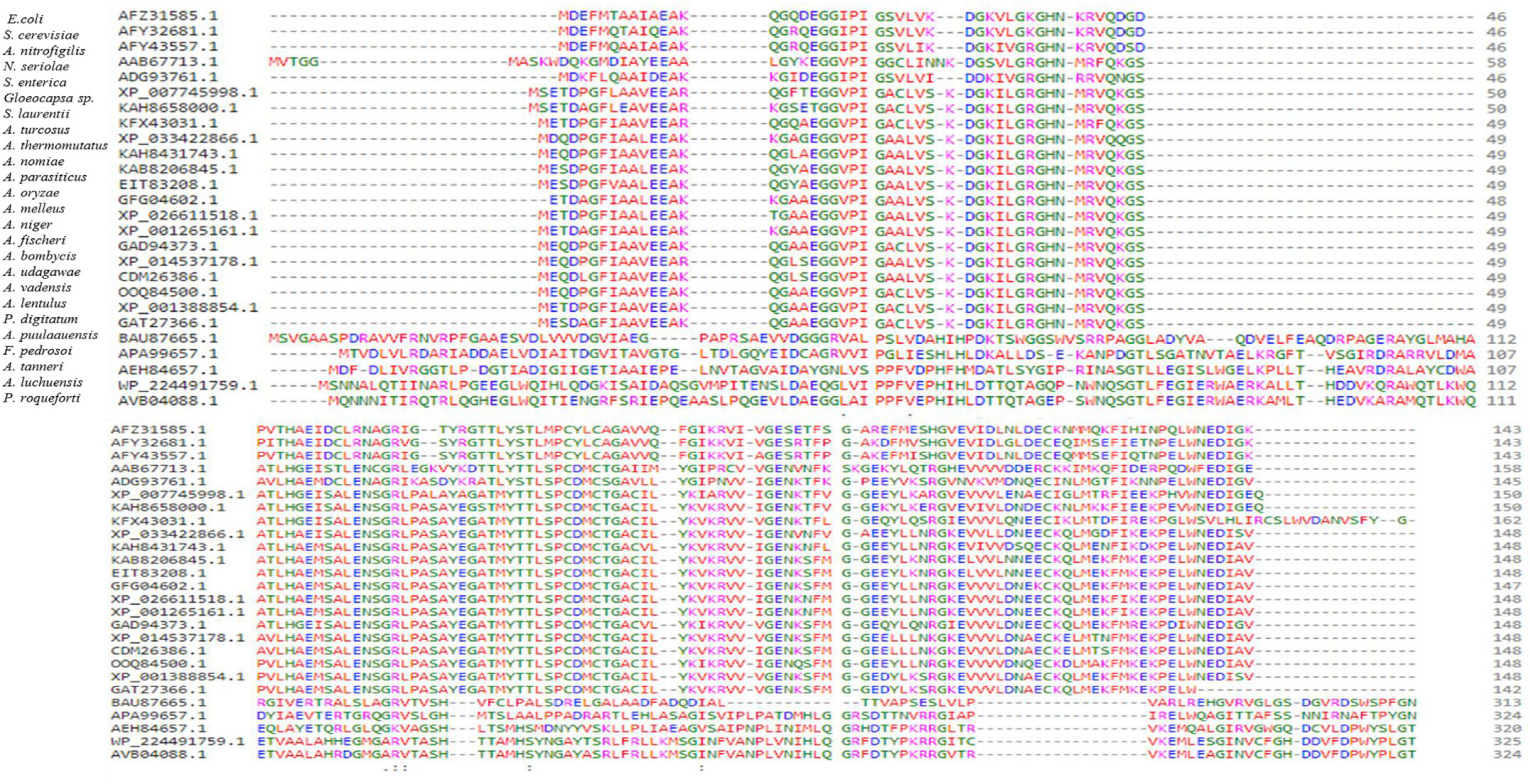
Amino acid alignment of CDA from various prokaryotes and eukaryotes, namely *E. coli, S. cerevisiae, A. nitrofigilis, N. seriolae, S. enterica, Gloeocapsa sp. S. laurentii, A. turcosus, A. thermomutatus, A. nomiae, A. parasiticus, A. oryzae, A. melleus, A. niger, A. fischeri, A. bombycis, A. udagawae, A. vadensis, A. lentulus, P. digitatum, A. puulaauensis, F. pedrosoi, A. tanneri, A. luchuensis* and *P. roqueforti.* The conserved residues are shown by colored asterisks. The homologue residues were shown with dots and colons.

**Figure 5:**
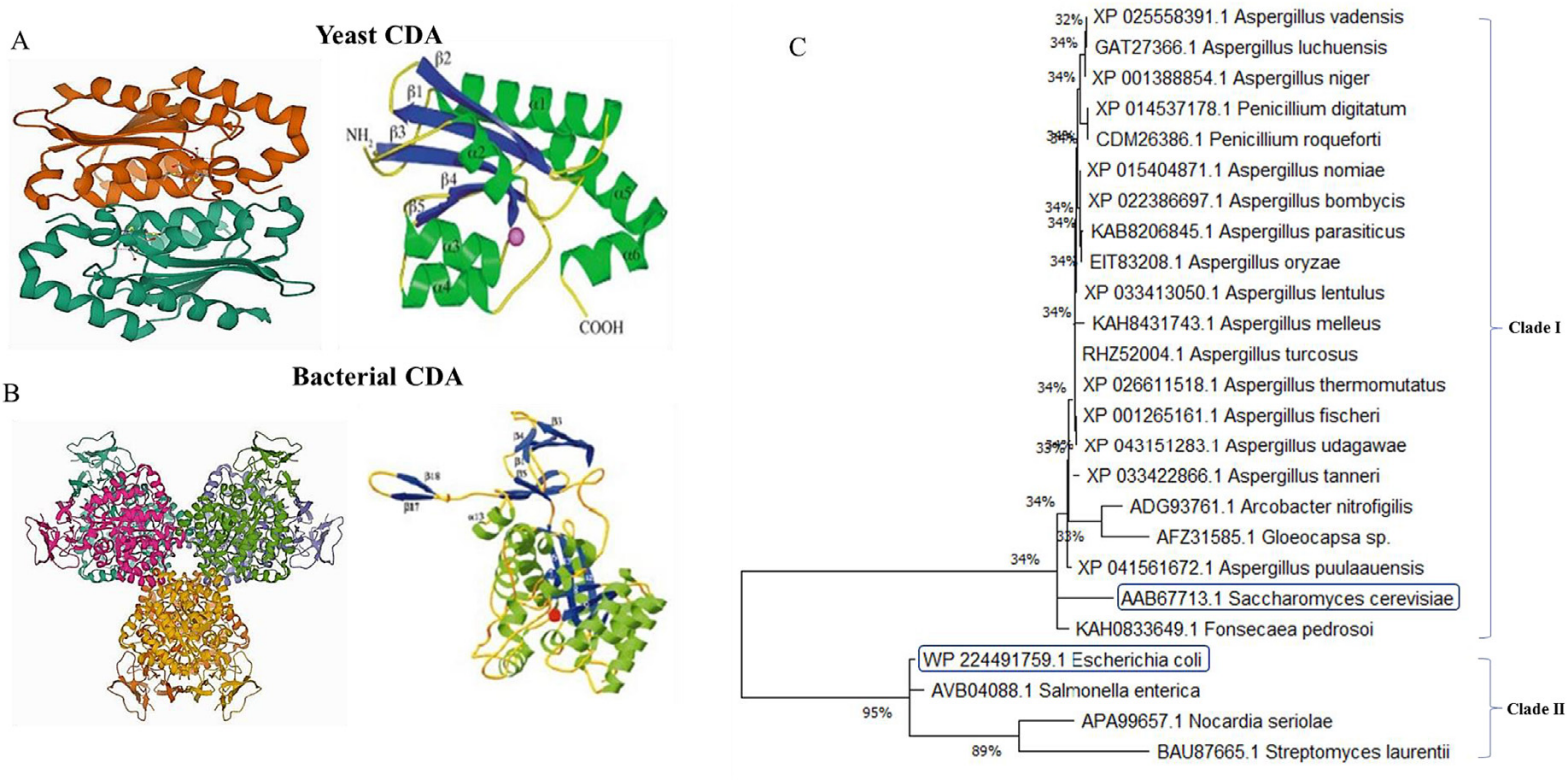
Crystal structures and phylogenetic analysis of CDA from different microorganisms based on their amino acids sequences. The stereo view of 3D structure of CDA from yeast (A) and *E. coli* (B). The three-layered α/β/α structure with a central β-shhets sandwiched on either sides by α-helices, as shown on right panel of A and B (Ireton et al. 2003; Ireton et al., 2002). C, Based on the amino acids sequences, from the phylogenetic analysis, two clades were evolved Clade I yeast CDA and Clade II *E. coli* CDA.

The crystal structure of yeast CDA has been characterized by X-ray crystallographic analysis (Ko et al., 2003). The protein structure contains five stranded β-sheets (β1–β5) with the strand order 2, 1, 3, 4, 5 and with β1 running antiparallel to the other strands. The β-sheets are sandwiched by two α-helices (αB-αD) on the other side (Ko et al., 2003; Yassin et al., 2022). The enzyme forms a tightly packed dimer in the crystal structure without significant differences between the two subunits. The dimer interface constituted by helical layer (αB-αD) and the C-terminal tail. The structural amino acids of CDA from different microorganisms were resolved, and the conserved regions were determined. Depending on the protein sequence data base, a comprehensive amino acid alignment was conducted to explore the structural and catalytic identities of CDA from different microorganisms using *E. coli* (WP224491759.1) and *S. cerevisiae* (AAB67713.1) as reference CDAs. The sequences of CDA were resolved from the National Center of Biotechnology Information (NCBI, http://www.ncbi.nih.gov). The alignment and the phylogenetic analysis were performed using ClustalW2 (Myers and Miller, 1988). The amino acids residues of CDA from different bacterial and fungal isolates were aligned. From the alignment profile of the amino acids sequence (Figure 4), the CDA sequences were categorized into clusters clade I, and clade II, representing the fungal and bacterial cluster, respectively. From the alignment profile, the conserved amino acids were D4, A10, E13, G17, E20, G21, G22, G22, G26, D33, K35, G38, H41, N42, R44, V45, Q46, H53, E55, L59, N61, G63, R64, Y70, C82, C85 and W142. From the phylogenetic tree, two clusters of CDA were clearly observed; clade I (Yeast CDA) and clade II (bacterial CDA) (Figure 5). The similarity of amino acid sequence of yeast CDA and bacterial CDA were approximated by about 35%. The amino acid sequence of CDA from *A. vadensis* (XP025558391.1), *A. luchuensis* (GAT27366.1), *A. niger* (XP001388854.1), *P. digitatum* (XP014537178.1), *P. roqueforti* (CDM26386.1), *A. nomiae* (XP015404871.1), *A. parasiticus* (KAB8206897.1), *A. oryzae* (EIT83208.1) displayed a significant proximity with *S. cerevisiae* CDA (AAB67713.1) (clade I) by about 95 %. While, the amino acids sequence of CDA from different bacterial isolates; *S. enterica* (AVB04088.1), *S. laurentii* (BAU87665.1) and *Nocardia seriolae* (APA99657.1) exhibited a similarity ratio 98% with the reference CDA of *E. coli* (WP224491759.1). Structurally, from the amino acid sequences of CDA and crystal structure, the conserved amino acids have been reported to be localized on the substrate binding active site domains and catalytically active domains of bacterial and fungal CDA, while, the other N-terminal and C-terminal domains were different, assuming the variations on the structural stability and catalytic efficiency of CDA from bacterial and fungal sources. The structural stability and catalytic efficiency of enzymes are strongly based on the N-terminal and C-terminal domains (Varland et al., 2015). N-terminal modifications of the nascent polypeptides derived from the ribosome for enzyme maturation is one of the most recognized posttranslational process involved in conformational stability of the enzymes (Varland et al., 2015). Methionine amino-peptidases is the universal initiator of methionine excision followed by a plethora of predicted modification process especially acetylation, propionylation, palmitoylation and ubiquitylation (Kerwar et al., 1971; Stock et al., 1987; Tooley and Tooley, 2014). The N-terminal domains are mainly related to the *in vivo* conformational and structural stability of proteins (Hwang et al., 2010; Tasaki et al., 2012; Kim et al., 2014). The fluctuation on the conformational structure and catalytic efficiencies of CDA from both bacterial and fungal sources might be attributed to the variation on the enzyme amino acid constitutions and conformational structures. The bacterial and fungal CDs are distinct from each other and have evolved separately. The 426-residue hexameric *E. coli* CDA had a similarity to adenosine deaminase that belongs to amidohydrolase superfamily, the four histidines and one aspartate located at similar spatial positions are conserved for metal coordination and enzyme catalysis (Ko et al., 2003). On the other hand, the 158-residue dimeric yeast CDA shares two conserved signature sequences “HXE and CXXC”, with deaminases, it has been grouped to the cytidine and deoxycytidylate deaminase family in the Pfam protein family (Bateman et al., 2002). The crystal structure of *E. coli* CDA reveals that the signature sequences contain a zinc binding motif, with histidine and two cysteines residues acting as zinc ligands while the glutamate serves as a proton shuttle (Betts et al., 1994).

### 1.3 Programmability of CDA for mediating the conversion of prodrug 5-FC into drug 5-FU

**Figure 6:**
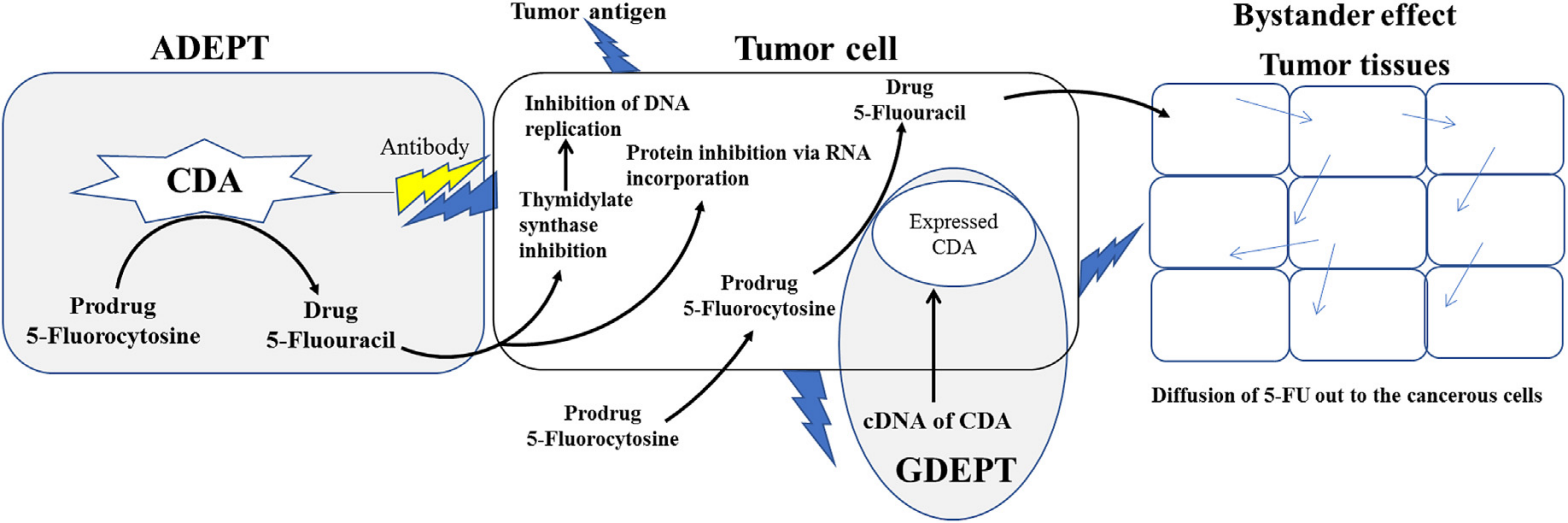
Antibody-directed Enzyme Prodrug Therapy (ADEPT) and Gene-Directed Enzyme Prodrug Therapy (GDEPT) as adapted from Xu and Mcleod, 2001).

The development of drug-resistant metabolic criteria to the current chemotherapeutic drugs is one of the main clinical limitations that halt this strategy, thus, the enzyme prodrug mediated therapy is one of the most recent emerged targeted/directed strategies that minimize the off-target effects of the traditional approaches. Cytosine deaminase-5-FC system is one of the remarkable recent approaches for targeting specific metabolic pathways in tumor cells. However, the enzyme catalytic efficiency and localization of the enzyme is the major challenge to the practical application of this system (Kogelberg et al., 2007; Sharma and Bagshawe, 2017). The non-specificity of cytotoxic agents and their effect on regeneration of normal cells is the major side-effect for most of traditional therapeutic approaches, thus restricting the drug to the tumor cells and decreasing the required dosage is major objective. Thus, generating an active cytotoxic drug from a non-toxic precursor only within or in close proximity to tumor is the main prospective. Localizing the effect of the cytotoxic drugs on sites of tumor cells by prodrug mediated CDA for minimizing the off-target effects of this system is the challenge for biotechnologist. Several strategies have been proposed for directing and localizing the system of CDA-5-FC towards the tumor cells namely; 1-Antibody-directed enzyme prodrug therapy (ADEPT)” in which the active enzyme can be delivered to the tumors cells *via* antibodies specific to tumor cells. 2- Gene-directed enzyme prodrug therapy (GDEPT), and Virus-directed enzyme prodrug therapy (VDEPT), in which the CDA coding genes was delivered to the target tumored tissues, followed by expression of the genes, subsequently the prodrug was provided, that sequentially activated the prodrug into active drug in the target tissues (Portsmouth et al., 2007; Tietze and Schmuck, 2011). There is a promising strategy in gene therapy, in which the combination between gene coding enzyme and a prodrug was occurred, the gene was delivered followed by their prodrugs. Additionally, expression of the CDA suicide gene *in vivo* was reported in the glioma tumor cell inducing DNA damage after addition of 5-FC, resulted in suppression of glioma cell proliferation (Chang et al., 2020). Implantation of the purified bacterial and fungal CDA in combination with 5-FC for prodrug mediating therapy of various tumor cells were frequently occurred (Michi et al., 2009). CDA is a non-human enzyme (Nishiyama et al., 1985; Katsuragi et al., 1987), however, the human cells possess cytidine deaminase which has the potency to convert 5-FC to the toxic form 5-FU (Hayden et al., 1998), through pyrimidine salvage pathway, that has been considered as the major drawback of this prodrug system. Due to the previous characters, CDA has been in suicide gene therapy (SGT) (Michi et al., 2009). Bystander effect is one of the main practical features of the prodrug mediated therapy, which is an essential factor in gene-prodrug system (Kuriyama et al., 1999) by dissemination to all the surrounding cancer cells. Several studies using CDA/5-FC, reporting the induction of bystander effect can be achieved by this system *in vitro* and *in vivo* (Huber et al., 1994; Kuriyama et al., 1995; Dong et al., 1996). The bystander effect is an important factor in success of suicide gene therapy because by which the cytotoxic effect can diffuse from cells (transduced) to neighboring cells (non-transduced) as shown in Figure 6. Due to the small size of the activated drugs, it can diffuse by concentration gradient towards the tumor cell, using this phenomena even if less than 10% of cells were transduce, it will be sufficient to influence on the cancer cells (Ardiani et al., 2012; Xiao et al., 2013). So, the spreading of toxic metabolites from cell (transduced) to cells (non-transduced) by active or passive diffusion is the main mechanism for the bystander effect (Karjoo et al., 2016).

### 1.4 Antibody-directed enzyme prodrug therapy (ADEPT)

Localizing the cytotoxic drug to the site of tumor cells by prodrug-enzyme mediated therapy, implementing the antibody-directed enzyme therapy approach, is the talented technology for minimizing the off-target side effects of the common chemotherapeutic drugs (Kogelberg et al., 2007; Sharma and Bagshawe, 2017). The main objective of ADEPT technology is to restrict the activity of drug to only the tumor sites, utilizing lower dosage of the drug, and avoiding the drug natural clearance, and thus increasing the therapeutic potential of drugs. The inadequate tumor specificity of cytotoxic agents by affecting on the normal cell renewal tissues is the key challenge for various traditional therapeutic approaches, thus restricting the drug to the tumor tissues cells and decreasing the required dosage. This can be attained by generating an active cytotoxic drug from a non-toxic precursor only within or in close proximity to tumor cells, thus guaranteeing that the active drug does not reach normal cell renewal tissues, via the plasma compartment, in dose limiting amounts. During the past 40 years, since the initiation of monoclonal technology, several trials have been proposed to improve the selectivity of cytotoxic drugs by conjugation with antibodies directed to tumor associated antigens (Köhler and Milstein, 1975; Baldwin and Byers, 1986). Similarly, biotoxins like diphtheria toxin and ricin (Moolten and Cooperband, 1970) requires internalization to be cytotoxic that usually directed by the target antibody. The concept of generating an active drug from inactive prodrug necessitates a catalyst, which should be non-human enzyme, completely absent in normal cells. The major challenge of this technology is the spontaneous activation of prodrugs (Paul et al., 1977) (Connors, 1978), in both tumor and normal cells due to the presence of the same enzymes or even isoenzymes with a catalytic efficiency on this prodrug. The concept of the Antibody Directed Enzyme Prodrug Therapy (ADEPT) approach had firstly reported by Philpott et al., (1973), with the main objective to selectively deliver the enzyme to only the cancerous sites without effecting on normal cells. The principle of ADEPT is 1) targeting the enzyme to the tumors by attaching it to antibody directed to a tumor associated antigen, 2) incorporation of the non-toxic prodrug, after clearance of the enzyme from the blood (Figure 6). The targeted enzyme converts the non-toxic prodrug into a potent cell killing drug within tumors to achieve their effective therapy without toxicity to the normal tissue. The principle of ADEPT is the conjugation of enzymes with the monoclonal antibodies that in turn binds to the *in vivo* tumor associated antigens, followed by subsequent addition of the prodrug. The active generated drug should reach tumor cells in lethal concentration without diffusing out to the normal tissues. To confine the localization of the cytotoxic agent to tumor sites, an effective conjugation of the antibody-enzyme conjugate (Ab-E) with the tumor sites other than normal sites, should be designed, prior administration of the prodrug. The phases of therapeutic efficiency of ADEPT could be categorized as follows; Initial phase in which a high concentration of Ab-E was applied for attaining the maximum binding of antibody with the target tumor sites (Rogers et al., 1986; Jain and Baxter, 1988; Yu et al., 1991). Clearance phase was to ensure the complete clearance of Ab-E, or inactivation of the enzyme in plasma and other normal tissues before the prodrug usage, that is necessary to minimize the prodrug activation at non-tumor sites and consequential toxicity. This involves inactivation and removal of enzyme from the blood and other non-tumor sites prior to prodrug administration, for avoiding the toxicity to normal tissue. To achieve this, numerous novel clearing agents in the form of glycosylated peptidomimetic molecules have been created, with the potency to bind with the enzymes active sites resulting in specific inhibition of the enzyme and subsequent clearance of the complex *via* the asialo-glycoprotein receptor (Bagshawe et al., 1994). The last phase is the addition of the prodrug. Although, the promising therapeutic potency of the ADEPT approach for minimizing the side effects of drug on the normal cells, the antigenicity of Ab-E, is the major challenge from the practical view.

Overall, the efficiency of the ADEPT depends on the following; 1- target antigen, 2- Choice of enzyme, and 3- Choice of antibody. Firstly, the target antigens should be localized on the surface of tumor cells “plasma membrane” to be more accessible sites for binding with the antibody component of Ab-E (Mason and Williams, 1980). The internalization of the Ab-E on the target cells authenticates the activation of the prodrug only on tumor cells. The target antigens might be a membrane bound or secreted antigens as targets for the antibody based therapy. Membrane bound antigens might be expected to confers longer dwell times for Ab-E than secreted antigens, while, the antigen density might be greater with secreted antigens (Gordon et al., 1982). Secreted antigens are usually present in the plasma, thus interference with the Ab-E might be occurred out of tumor cells (Begent et al., 1980). Thus, selecting of specific antigen for each type of cancer cells might be the proper approach to minimize the off-target effect of antibody. Overexpressed gene products with an external domain on the cell membrane (Park et al., 1992), may be a viable targets, in addition to the growth factor receptors (Perez-Soler et al., 1992). Thus, heterogeneity in the antigen distribution within the population of cancer cells, and the potential to overcome this challenge by specific monoclonal and polyclonal antibodies has been extensively considered (Bagshawe, 1987; Senter et al., 1988).

Secondly, choice of enzyme, the potential enzymes for this approach should be characterized by unique biochemical properties. For example, the optimum enzymatic activity should be close to the pH of tumor extracellular matrix. The catalytic efficiency and affinity of the enzyme should be very high to minimize the required enzyme with higher affinity to the target prodrug substrate. Additionally, the enzyme dependence on cofactor and coenzyme should be minimized to reduce the administrated components. Several enzymes have been used for mediating the prodrugs by ADEPT technology as listed in Table 3. For example, carboxypeptidase G2 (CPG2) is a non-human enzyme, catalyze of the activation of pro-methotrexate anticancer prodrug (Sherwood et al., 1985), this enzyme has been cloned from *Pseudomonas* sp, nevertheless, the enzyme antigenicity was the major disadvantage that limits its application. Additionally, presence of isoenzyme homologs in human tissues and plasma, similar to the applied target enzyme, is one of the major tackles that halt the progress on this technology. In terms of specificity, enzymes from non-human sources appear more affordable and their immunogenicity might be controllable via conjugation with natural innate biocompatible polymers such as polyethylene glycol (El-Sayed et al., 2014, 2015, El-Baz et al., 2018, 2019; ​El Sayed and El-Sayed, 2020a, ​El Sayed and El-Sayed, 2020b). Thirdly, choice of antibody, the first available monoclonal antibodies (W14 and SB 10) to human chorionic gonadotrophin (PhCG) and A5B7 to CEA had been reported (Bagshawe et al., 1994). Several antibodies have been used in ADEPT including human carcinoma associated antigens (Senter et al., 1988), lymphoma (Senter et al., 1988), ovarian carcinoma (Bagshawe et al., 1994), placental alkaline phosphatase (Neuberger et al., 1984) and melanoma (Neuberger et al., 1984). High affinity monoclonal antibodies directed at tumor associated antigens appear to be essential for the ADEPT approach. Bivalent antibodies may be better than univalent in terms of dwell time. The small molecules are usually penetrates tumor cells easily, with desirable rapid blood clearance Jain and Baxter, 1988; Larson (1990); Yu et al., 1991). Conjugates of the enzyme-antibody were prepared by thiolating the amino groups of antibody fragment with 5-5-acetylthioglycolic acid N-hydroxy-succinimide ester and coupling via physiologically stable thioether bond to the enzyme maleimide groups (Searle et al., 1986; Melton et al., 1993).

**Table 3:** Different systems of ADEPT and GDEPT.

Enzyme	Prodrugs	Drugs	Action	Bystander Effect	References
Cytosine deaminase	5-Fluorocytosine (5-FC)	5-Fluorouracil (5-FU)	Inhibit thymidylate synthetase So, blocks the synthesis of both DNA and RNA, usually affect the dividing cells but at high concentration inhibit both dividing and non-dividing cells.	High	(Hanna et al., 1997)
					(Johnson et al., 2011)
Purine nucleoside phosphorylase	6-Methylpurine deoxyriboside	6-methylpurine	Inhibits all of DNA, RNA and protein synthesis	High	(Lockett et al., 1997)
					(Martiniello-wilks et al., 1998)
					(Mohr et al., 2000)
Nitroreductase	CB1954 and analogs	5-(Aziridin 1-yl) 4-hydroxylamino 2-nitrobenzamide	Cross linker agent in interstrand of DNA; effect on both dividing and non-dividing cells	Very high	(Jaberipour et al., 2010)
Herpes simplex virus thymidine kinase	Ganciclovir (GCV)	Ganciclovir monophosphate (GCV-TP)	Metabolized to triphosphate nucleotide; prevent DNA synthesis by inhibiting DNA polymerase	High	(Moolten, 1986)
			Active in dividing cells		(Nicholas et al., 2003)
Cytochrome P450	Oxazaphosphorines: cyclophosphamide	4-Hydroxycyclophosphamide	Cross linker agent in interstrand of DNA	Medium	(Chen et al., 1997)
					(Chen and Waxman, 2005)
Carboxypeptidase G2	Nitrogen mustard CMDA	Phenol-bis-iodo nitrogen	Cross linker agent in interstrand of DNA	High	(Hedley et al., 2007)
		Mustard CMBA			
Carboxyesterase	Irinotecan (CPT11)	SN38 (camptothecin)	Binds to DNA topoisomerase I so, breaks DNA into single strands.		(Wierdl et al., 2008)

### 1.5 Gene-directed enzyme prodrug therapy (GDEPT)

The strategy of gene-directed enzyme prodrug therapy (GDEPT) had been proposed to overcome the challenges and side effects of the traditional chemotherapy (Karjoo et al., 2013; Yuan and Baxter, 1991). The mechanism of selective targeting the uracil pathway in tumor cells than normal cells was firstly explored in 1957 (Heidelberger et al., 1957). In 1970s, a new era of targeted therapy has been emerged with the appearance and conjugation of monoclonal antibodies with cytotoxic compounds (Chabner and Roberts, 2005; DeVita and Chu, 2008, Eastman and Perez, 2006). GDEPT is one of novel strategies in cancer therapy by selective transferring of target gene that catalyzes the production of toxic drug from a nontoxic prodrug (Springer and Niculescu-Duvaz, 2000; Portsmouth et al., 2007) (Figure 6). The GDEPT strategy involves two steps, 1-Transduction of gene coding the harmless enzyme into the tumor cells, 2- Mediating the nontoxic prodrug to be converted into toxic metabolites resulted in cell death (Aghi et al., 2000; Hajri et al., 2004; Portsmouth et al., 2007). This approach pledge the efficiency of anticancer drug and decreases its side effects on normal cell that, with potential increase to the therapeutic indexes over the conventional strategies “radiotherapy or chemotherapy” (Karjoo et al., 2013). This strategy provides two unique advantages over the conventional ones, firstly, the suicide gene expression is mainly depends on a specific promoter in the tumor cell, so the expression of this gene can be selectively done in only the tumor cell, but not in normal cells (Saukkonen and Hemminki, 2004; Dorer and Nettelbeck, 2009). This character give the chance to the prodrug to be activated only in the cancerous cell reducing the possibility of off-target toxicity (Yao et al., 2011), leading to a higher concentration of the toxic compound in the target tumor cell over the normal cells (Saukkonen and Hemminki, 2004). Secondly, bystander effect is one of the most potent features that confirms the success of GDEPT over other conventional strategies, allowing the diffusion of cytotoxic effect of drug from the transduced tumor cell to the other neighboring non transduced cells by passive or active diffusion (Karjoo et al., 2013, 2016).

In GDEPT, the enzymes can be classified into two groups: (a) exogenous group; which usually originates from microorganisms as bacteria or viruses and has no counterpart in human, like cytosine deaminase and thymidine kinase, and (b) endogenous group; which can be found in human normal cell like cytochrome P450 (Niculescu-Duvaz and Springer, 2005; Portsmouth et al., 2007). Opposite to the second set, the first set might be likely to be immunogenic with time (Shalev et al., 2000; Karjoo et al., 2013). Despite the second set is less to be immunogenic, it may activate the prodrug in normal cells, producing a lot of side effects (Niculescu-Duvaz and Springer, 2005). The efficiency of suicide gene (transduced gene)/prodrug strategy mainly depends on activity of the transduced gene/enzyme. It should be absent in human, non-toxic to the normal cell, displaying a high catalytic efficiency towards prodrug at low concentration (low *K*_*m*_ and high *K*_*cat*_), smaller molecular mass to be more easily expressible in the expression vectors with higher ability to fully activate the prodrug independent on other factors. Prodrug should be capable of penetrating the cancerous cell and diffuse through it, easily to be activated by the enzyme with powerful bystander effect with long half-life time (Lammers et al., 2012). Generally the systems of gene delivery could be microorganisms such as virus, bacteria and yeast, or dendritic or stem cells (Mohit and Rafati, 2013) and synthetic vectors (polymeric and lipid based) (Helen et al., 2010; Karjoo et al., 2016). Several enzyme prodrug systems have been used in GDEPT are summarized in Table 3 (Karjoo et al., 2013, 2016). Vaccinia virus with the prodrug activator gene, might be used as a tool for gene delivery, for augmenting the antitumor efficiency of target prodrug, combined with the effect of vaccinia virus and chemotherapy together (Ding et al., 2020). 5-FC is an orally bioavailable FDA approved antifungal drug with efficiency to crosses the blood-brain barrier (Takahashi et al., 2014). CDA armed Vaccinia virus in conjugation with subsequent 5-FC, provides a direct toxicity killing of tumor cells by local production of 5-FU. Vaccinia virus induced a local and systemic immunotherapeutic response resulting in long-term survival after cessation of 5-FC treatment (Yagiz et al., 2016; Hiraoka et al., 2017). Yeast CDA gene has been recently cloned and overexpressed in *Vaccinia virus* VG9, displaying a higher activity in conversion of non-toxic prodrug 5-FC into toxic drug 5-FU (Ding et al., 2020).

### 1.6 Rationality of CDA/5-fluorcytosine system

The 5-fluorouracil drug had been extensively used in treatment of various types of cancer cells, however, their cytotoxicity to non-cancerous tissues, undesirable effects includes diarrhea, and toxicity to cardiac tissues (Papanastasopoulos and Stebbing, 2014) are the main limiting factors for broad-range application of this drug. The antimetabolite 5-fluorouracil has been produced by the conversion of non-toxic prodrug 5-fluorocytosine by the action of bacterial or fungal CDA, which is a non-human enzyme (Duarte et al., 2012). The drug 5-FU has been characterized by a small size that enables it to diffuse rapidly into/out the neighboring cells resulting in a bystander effect (E. Kievit et al., 2000). The intracellular enzymes convert 5-FU to different metabolites, which finally inhibits thymidylate synthase and causing cell death (Figure 2). Because 5-FC can diffuse through blood brain barrier, this advantageous property can be used in treating tumors such as glioblastoma cancer (Ostertag et al., 2012). 5-FU is a radiosenseitizer chemotherapeutic agent, that can be used in the same time with ionizing radiation *in vivo* and *in vitro* which can enhance tumor killing activity (Hanna et al., 1997; Kaliberov and Buchsbaum 2012). However, CDA/5-FC system has a few drawbacks, for example gut normal flora could metabolize 5-fluorocytosine and produce 5-fluorouracil causing some undesirable effects (Karjoo et al., 2016). Bacterial CDA from *E. coli* displaying an efficient activity towards various tumor cells, however, CDA from yeast displayed a feasible kinetic properties for 5-FC (Johnson et al., 2011). Unfortunately, yeast CDA had a lower thermal stability, thus, a mutant of yeast CDA was created with new features like structural/thermal stability with a higher potency of cancer cells to sense 5-FC treatment comparing to normal yeast CDA (Korkegian et al., 2005; Stolworthy et al., 2008). A new recombinant oncolytic herpes simplex virus type 1 (oHSV-1) armed with *E. coli* CDA in combination with the prodrug 5-FC was constructed, displayed a strong efficacy against melanoma cell lines (Houtzagers et al., 2020; Liu et al., 2020). Also, combination of CDA and the prodrug 5-FC loaded on chitosan-silver nanoparticle displayed a strong activity in treatment of human breast carcinoma cell line and other solid tumors (Horo et al., 2020). Because of its ability to convert the relatively nontoxic 5-fluorocytosine (5-FC) into 5-FU and its absence in mammalian cells, CD has become an attractive candidate for the reduction of 5-FU toxicity toward normal distal tissues in enzyme-prodrug gene therapy (Greco and Dachs, 2001). Several studies demonstrated that yeast CD significantly had a higher therapeutic efficacy, than *E. coli* CDA, that might be due to the higher efficiency of the 5-FC into 5-FU by the yeast CD (Kievit et al., 1999; Kievit et al., 2000). Yeast CD, therefore, appears to be a better candidate for gene therapy.

### 1.7 Protein modification by cross-linking with immunogenically innate polymers

Several approaches have been explored to improve the kinetic properties and stabilizing the catalytic structural orientation of enzymes for *in vivo* applications. The specificity and selectivity, lower toxicity to normal tissues, solubility formulation, and delivery route to the target tissues, optimum dose and immunogenicity are the common challenges (El-Sayed et al., 2015a, El-Sayed et al., 2015, El-Sayed et al., 2015b, El-Sayed et al., 2015c, El-Sayed et al., 2016, El-Sayed et al., 2020). So, formulation of proteins by cross linking/conjugation to polymers to enhance its activity and pharmacokinetics properties has been recognized as one of the most applicable approach (Pasut, 2014). Several naturally innate polymers such as polyethylene glycol, chitosan, and dextran have been implanted for conjugation of therapeutic enzymes for improving their therapeutic potency such as structural stability, catalytic efficiency, protection from the *in vivo* proteolytic cleavage, increasing the half-life times of the target drugs (El-Sayed et al., 2014, El-Sayed et al., 2015a, El-Sayed et al., 2015, El-Sayed et al., 2015b, El-Sayed et al., 2016, El-Sayed et al., 2019). Conjugation of enzymes with polyethylene glycol (PEGylation) strongly improves the enzyme pharmacokinetic properties, reducing antigenicity, and stabilizing the conformational structures of enzymes (Zhang et al., 2015; El-Baz et al., 2011a,b, El-Baz et al., 2018). Dextran has been frequently authenticated as non-immunogenic, biocompatible, biodegradable compound, and stabilizer for the conformational structures of various therapeutic enzymes (El-Sayed et al., 2014, 2016; Badr et al., 2021; Raafat et al., 2021). Dextran is a natural polysaccharide of about 95% glycosidic linkages at position α-1,6 and 5% at 1,3-linkages that has been produced by *Leuconostoc mesenteroids* and *Streptobacterium dextranicum* (Varshosaz, 2012). The presence of 95% linear linkages in dextran polymer makes it a water soluble molecule and hence, enzyme conjugation with dextran increase the water hydrophilic identity of the enzyme in addition to shielding the antigenic sites of the enzymes, thus, protecting the enzymes antigenic reactions that in turn increases the half-life of enzymes *in vivo* (Murakami et al., 2003; Vertommen et al., 2005; El-Baz et al., 2017, ​El-Ghareeb et al., 2012). The unique chemical features of dextran especially higher hydrophilicity, frequency of glycosidic bonds which increases the higher stability in alkaline and acidic environments, protecting the conjugated enzyme from biodegradation polymer, afford this polymer to be the optimum carrier in drugs delivery systems (Varshosaz, 2012). Several studies confirming the efficiency of dextran as successful carrier in delivery of methotrexate (Dang et al., 1994), 5-fluorouracil (Hao et al., 2006), L-arginine deiminase, L-methionine γ-lyase, peptidyl arginine deiminase, cystathionine γ-lyase, homocysteine γ-lyase and arginase (El-Sayed et al., 2014, El-Sayed et al., 2015a, El-Sayed et al., 2015, El-Sayed et al., 2015b).

### 1.8 Future perspectives of therapeutic potency of cytosine deaminase

Combinatorial usage of CDA with the prodrug 5-fluorocytosine as an efficient strategy for cancer therapy seems to be the promising approach. However, enzyme antigenicity, targetability, and side effects are the most challenges that limits the auxiliary applications of this technology (Springer and Niculescu-Duvaz, 2000; Khan et al., 2017). Thus, enzymes with higher stability, catalytic affinity to the prodrug, less antigenicity being the most feasible objective for successfulness of this approach (Encell et al., 1999; Patel et al., 2016). Screening for CDA from novel fungal isolates with unique biochemical properties might be one of the most practical ways for improving the efficiency of this approach. Fungi have been considered as a repertoire for novel enzyme with higher human compatibility.

## 2 Conclusion

Cytosine deaminase (CDA) mediating the conversion of non-toxic prodrug 5-fluorcytosine (5-FC) into toxic drug 5-fluorouracil (5-FU) has been recognized as a powerful cancer-therapeutic approach comparing to the traditional chemotherapies and radiotherapies. 5-FU has been approved as a prominent anticancer drug, however, the main challenge of this approach are the short half-life, lack of selectivity and emergence of the drug resistance. Thus, mediating the 5-FU to the tumor cells by CDA is one of the most feasible approaches to direct the drug to the tumor cells, reducing its toxic effects and improving their pharmacokinetic properties. However, the efficiency, stability, antigenicity and targetability of CDA-5-FC, are the challenges that limit the clinical applications of this approach. Thus, exploring the biochemical properties of CDA, and the different approaches for localizing the CDA-5-FC system to the tumor cells via the antibody directed enzyme prodrug therapy (ADEPT) and gene directed prodrug therapy (GDEPT) had emphasized on this review. To the knowledge, this is the first report unraveling the perspectives for increasing the therapeutic efficacy, and targetability of the CDA-5-FC system for the further clinical approaches.

## Declarations

### Author contribution statement

All authors listed have significantly contributed to the development and the writing of this article.

### Funding statement

The work was supported by the Egyptian Academy of Scientific Research and Technology (ASRT-2022).

### Data availability statement

Data included in article/supplementary material/referenced in article.

### Competing interest statement

The authors declare no conflict of interest.

### Additional information

No additional information is available for this paper.
